# Ontario Veterinary Medical Association

**Published:** 1887-01

**Authors:** 


### ONTARIO VETERINARY MEDICAL ASSOCIATION.

The annual meeting of this association was held in Toronto, December 21st, 1886. There was a good attendance of members, the large lecture room of the veterinary college giving the accommodation necessary. Major Loyd, V.S., president of the association, occupied the chair, and opened the proceedings by an excellent address, passing in review many matters of interest to the members.

After routine business, J. Wende, V.S., of Buffalo, N. Y., was called upon, and read a paper on the Etiology of Schirrous Cord, Abscess and Fistula of Scrotum. Many theories have been put forward to account for this sequel of castration (Schirrous cord). Among others, Professor Johne, of Dresden, claims to have discovered a microbe in such growths and holds this micro-organism to be the essential cause. Professor Williams holds it to be due to a failure to separate the cord from its adhesions when the cord is removed, etc. The essayist, however, considers that none of the theories yet published will really account for champignon. The cause is, any foreign body, as blood, pus, etc., being contained in a sac of the serous covering, which sac is produced by the retraction of the cremaster muscle, when enough of the tunica vaginalis is not removed. This he discovered accidentally, while dissecting a case of Schirrous cord. In order to prove this theory, twelve colts were operated on, the cord on one side of several being operated on properly, in the others the sac above spoken of was purposely produced and some foreign body introduced, but of the sixteen cords so treated, fourteen cases of champignon resulted, and two cases of abscess. A spirited discussion resulted, which was taken part in by Messrs. Elliott, Duncan, Shaw, Granside, Hawkins, Gibb, Cowan and others. The opinion was expressed that much credit was due to the essayist for his bold experiments, which experiments would almost certainly injure his local reputation and standing. To such experiments, progress in science is often due. On motion the association passed a hearty vote of thanks to Mr. Wade. J. Hawkins, V.S., of Detroit, Mich., gave an interesting account of his experience with sulphate of eserine, or physostigma. He had found it of extreme value in cases of constipation, with flatulence. It is not so valuable in acute indigestion. A watery solution is used, the dose being half a grain to one grain, given hypodermically, the dose being much larger than is recommended in books. Sulpho carbolate of soda, in doses of four drachms, was the best method of treating the distention found in acute indigestion. The speaker would be happy to obtain the drug (eserine) for those who had not used it.

J. F. Smith uses it by the intra venous method of injection. He agrees as to the slight value of the drug in acute indigestion.

Mr. Quinn gave a short account of his method of operating on cryptorchids, and promised a paper on the subject for the next meeting.

A lively discussion then ensued in regard to proposed legislation for the better protection of the Veterinary Profession in Ontario.

The association resolved that registration of all Veterinary Surgeons be insisted upon, and that no one who is not so registered shall be permitted to practice "for hire, gain or hope of reward." A committee will wait upon the Agricultural and Arts Association to ask their consent to these changes in their Act, and to press the matter upon the attention of the House of Assembly.

The election of officers was then proceeded with, resulting as follows: Honorary President, Professor Smith, F. R. C. V. S., and principal of the Ontario Veterinary College; President, W. Shaw. V. S., Dayton, Ohio; 1st Vice-President, T. C. Grenside, V. S.; 2d Vice-President, J. F. Quinn, V. S.; Secretary, C. A. Sweetapple, V. S.; Treasurer, W. Cowan, V. S., and a Board of eight Directors.

